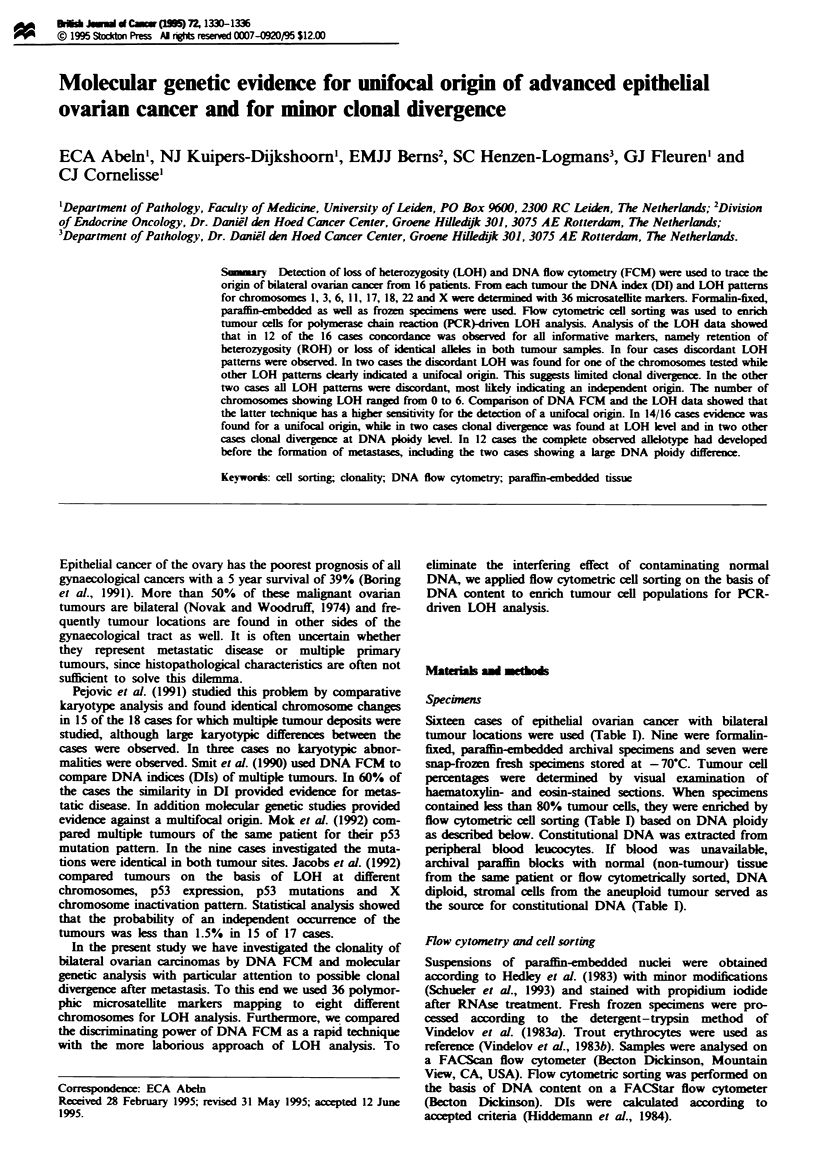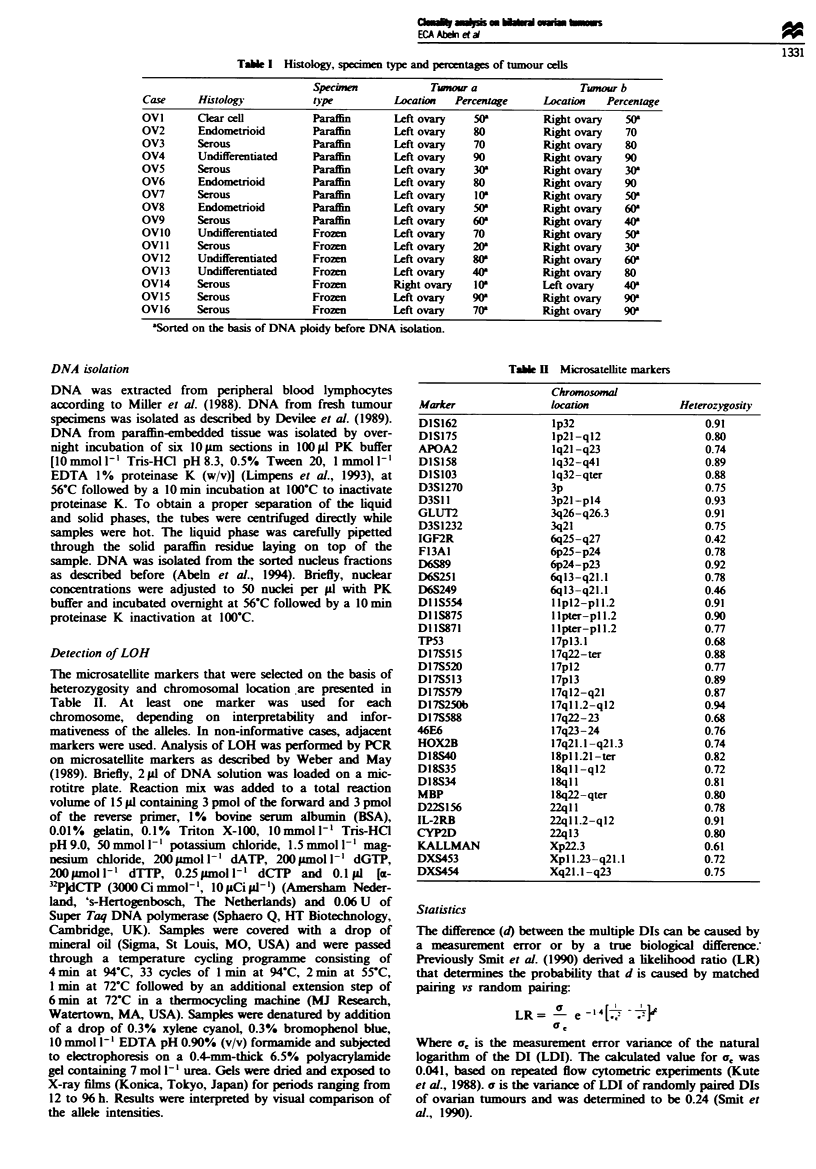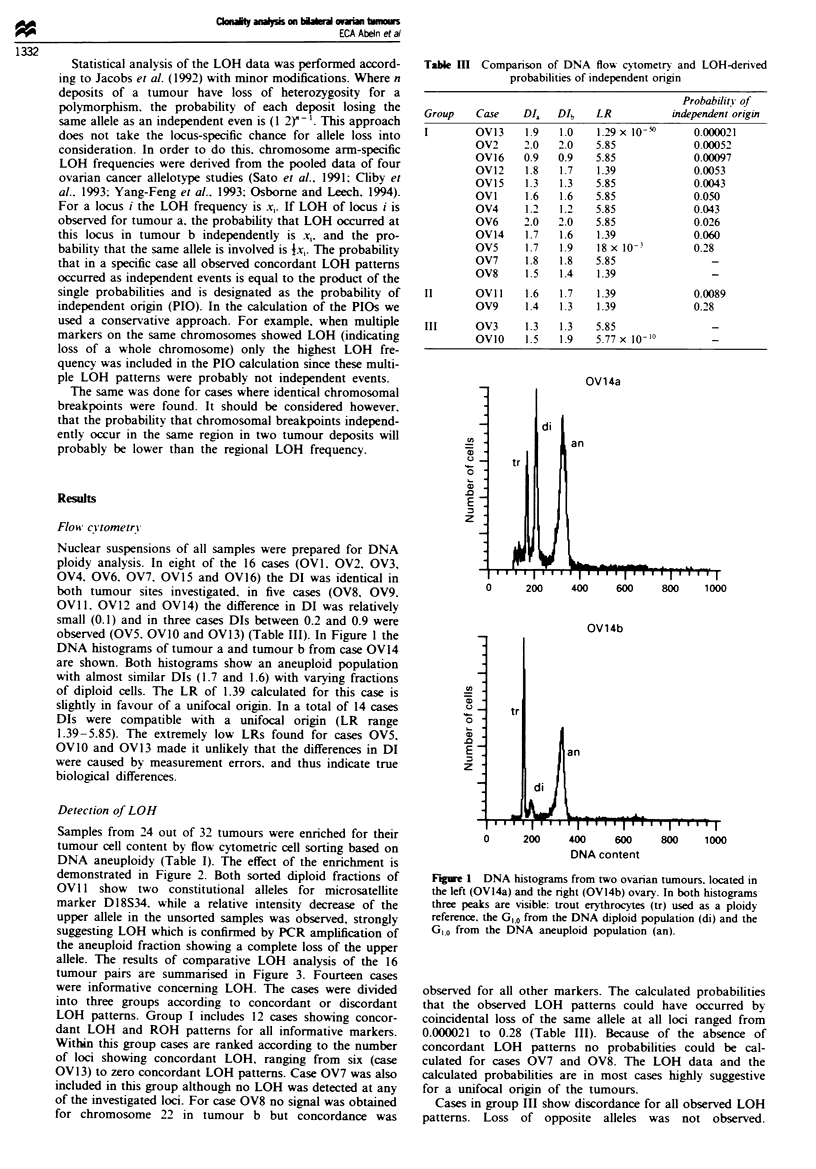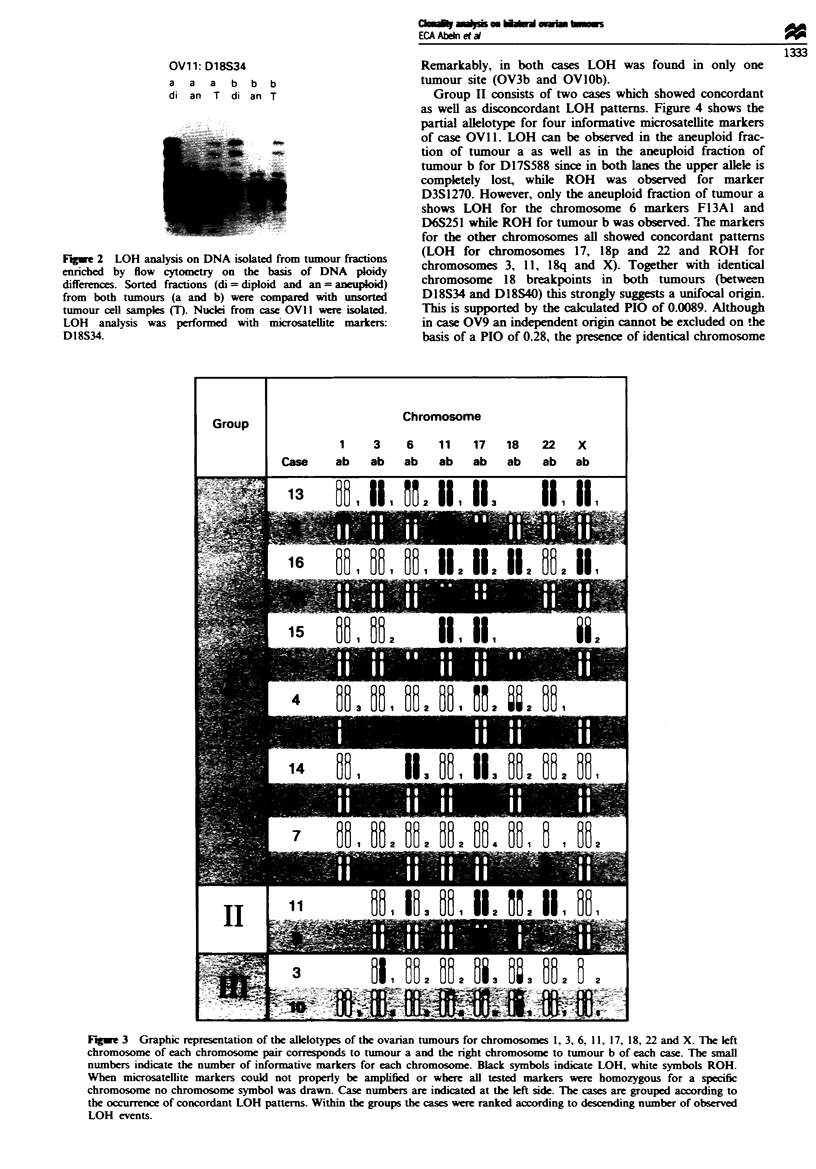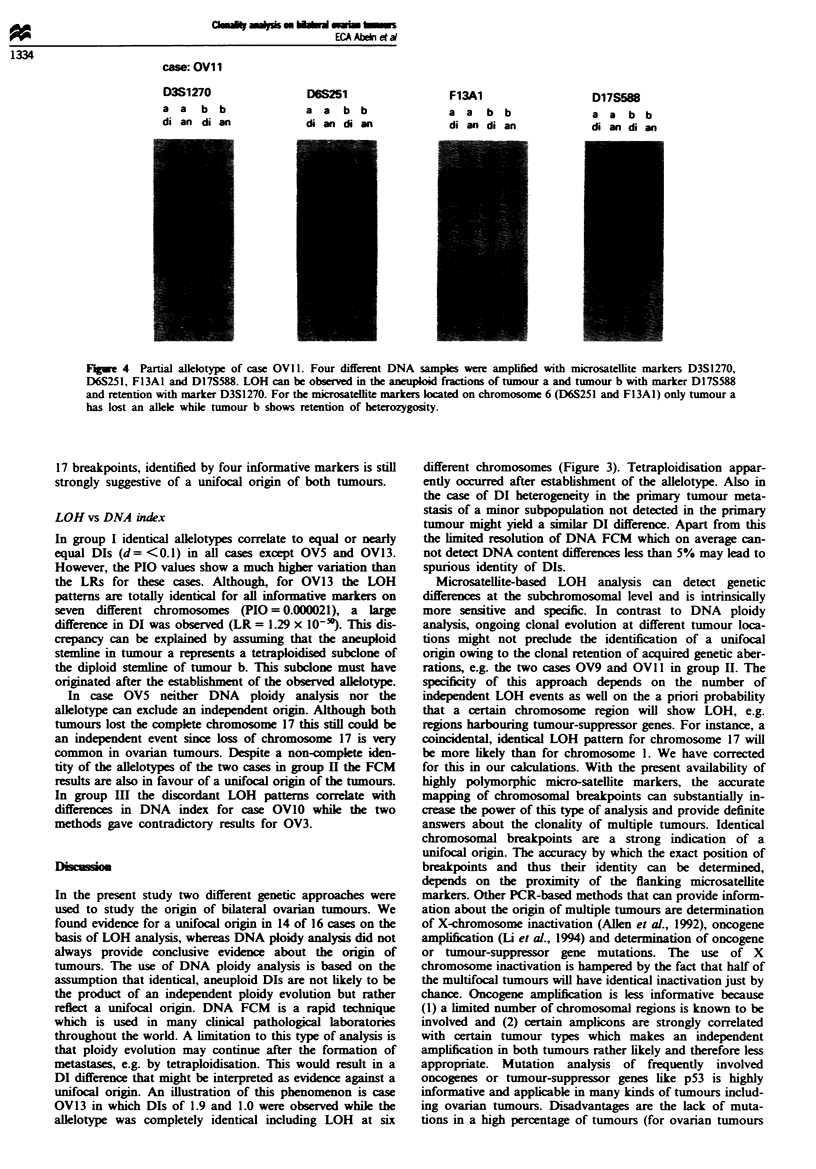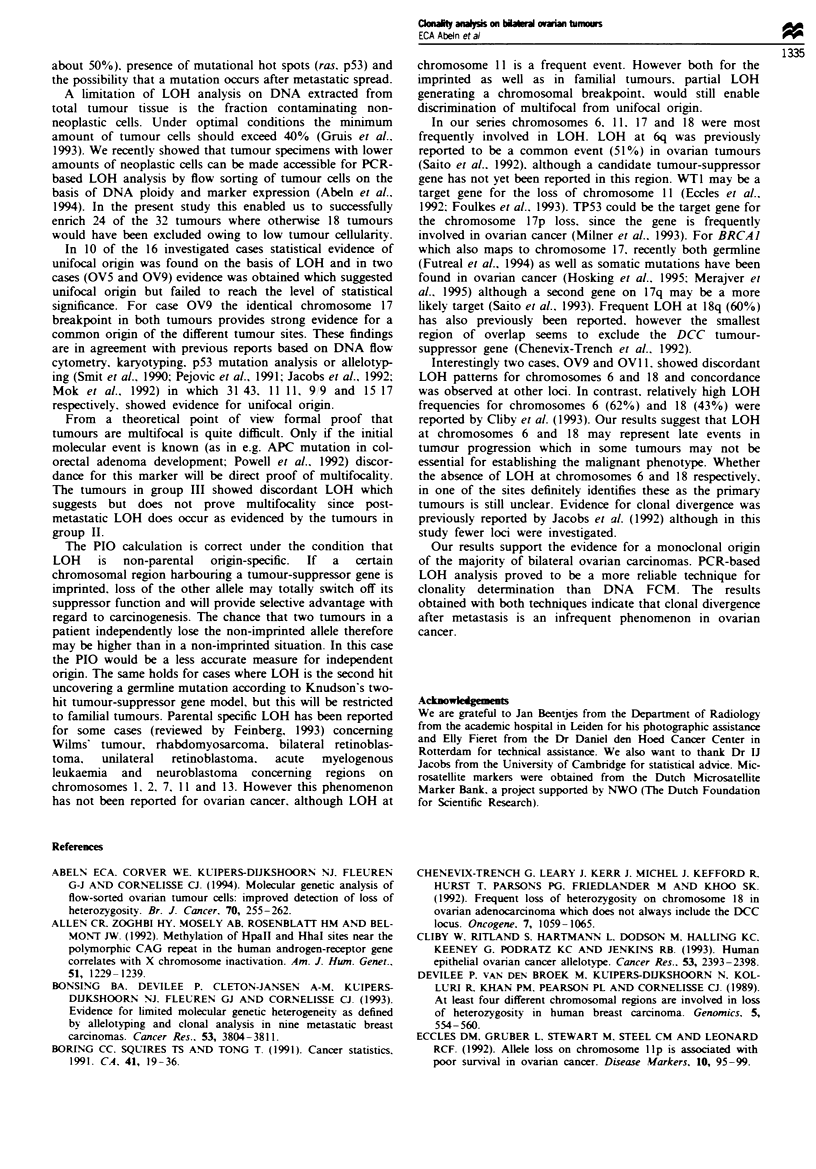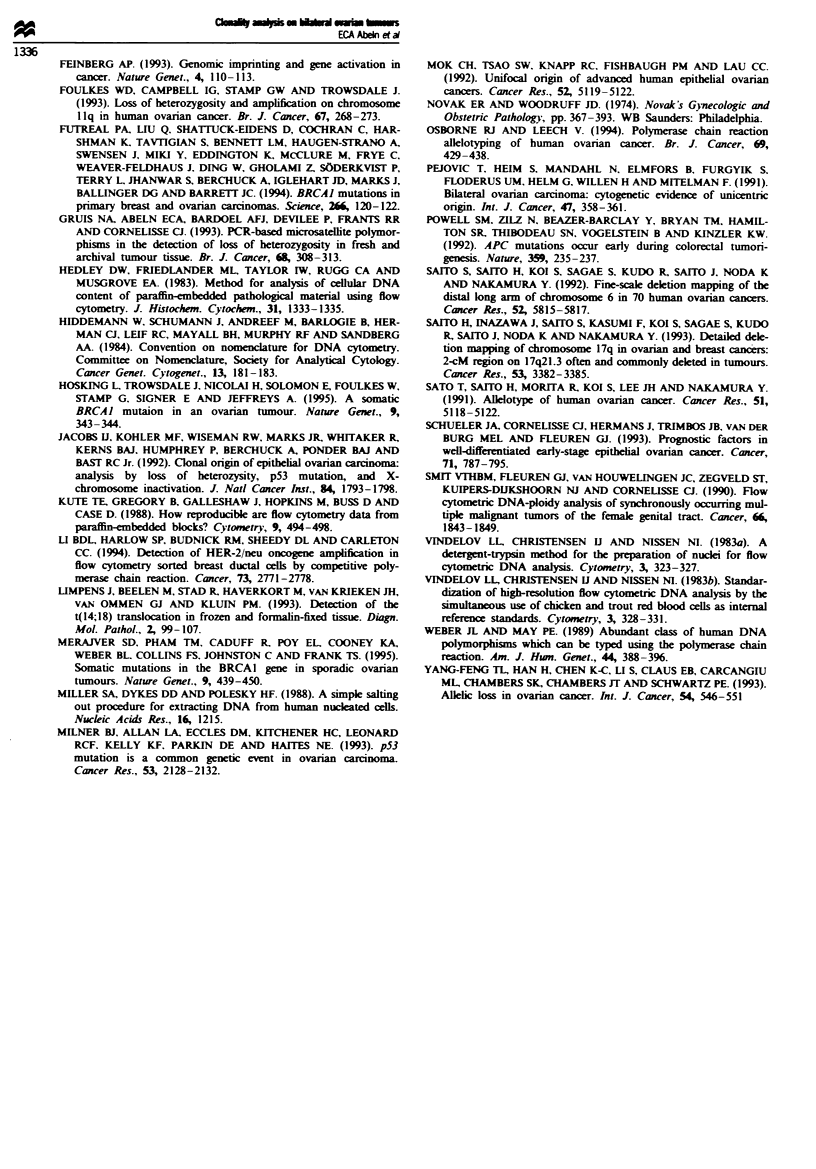# Molecular genetic evidence for unifocal origin of advanced epithelial ovarian cancer and for minor clonal divergence.

**DOI:** 10.1038/bjc.1995.510

**Published:** 1995-11

**Authors:** E. C. Abeln, N. J. Kuipers-Dijkshoorn, E. M. Berns, S. C. Henzen-Logmans, G. J. Fleuren, C. J. Cornelisse

**Affiliations:** Department of Pathology, Faculty of Medicine, University of Leiden, The Netherlands.

## Abstract

**Images:**


© 1995 Stockton Press All rights reserved 0007-0920/95 $12.00

### Molecular genetic evidence for unifocal origin of advanced epithelial ovarian cancer and for minor clonal divergence

ECA Abeln[1], NJ Kuipers-Dijkshoorn[1], EMJJ Berns[2], SC Henzen-Logmans[3], GJ Fleuren[1] and CJ Cornelisse[1]

[1]*Department of Pathology, Faculty of Medicine, University of Leiden, PO Box 9600, 2300 RC Leiden, The Netherlands;* [2]*Division of Endocrine Oncology, Dr. Daniël den Hoed Cancer Center, Groene Hilledijk 301, 3075 AE Rotterdam, The Netherlands;* [3]*Department of Pathology, Dr. Daniël den Hoed Cancer Center, Groene Hilledijk 301, 3075 AE Rotterdam, The Netherlands.*

**Summary** Detection of loss of heterozygosity (LOH) and DNA flow cytometry (FCM) were used to trace the origin of bilateral ovarian cancer from 16 patients. From each tumour the DNA index (DI) and LOH patterns for chromosomes 1, 3, 6, 11, 17, 18, 22 and X were determined with 36 microsatellite markers. Formalin-fixed, paraffin-embedded as well as frozen specimens were used. Flow cytometric cell sorting was used to enrich tumour cells for polymerase chain reaction (PCR)-driven LOH analysis. Analysis of the LOH data showed that in 12 of the 16 cases concordance was observed for all informative markers, namely retention of heterozygosity (ROH) or loss of identical alleles in both tumour samples. In four cases discordant LOH patterns were observed. In two cases the discordant LOH was found for one of the chromosomes tested while other LOH patterns clearly indicated a unifocal origin. This suggests limited clonal divergence. In the other two cases all LOH patterns were discordant, most likely indicating an independent origin. The number of chromosomes showing LOH ranged from 0 to 6. Comparison of DNA FCM and the LOH data showed that the latter technique has a higher sensitivity for the detection of a unifocal origin. In 14/16 cases evidence was found for a unifocal origin, while in two cases clonal divergence was found at LOH level and in two other cases clonal divergence at DNA ploidy level. In 12 cases the complete observed allelotype had developed before the formation of metastases, including the two cases showing a large DNA ploidy difference.

**Keywords**: cell sorting; clonality; DNA flow cytometry; paraffin-embedded tissue

Epithelial cancer of the ovary has the poorest prognosis of all gynaecological cancers with a 5 year survival of 39% (Boring *et al.*, 1991). More than 50% of these malignant ovarian tumours are bilateral (Novak and Woodruff, 1974) and frequently tumour locations are found in other sides of the gynaecological tract as well. It is often uncertain whether they represent metastatic disease or multiple primary tumours, since histopathological characteristics are often not sufficient to solve this dilemma.

Pejovic *et al.* (1991) studied this problem by comparative karyotype analysis and found identical chromosome changes in 15 of the 18 cases for which multiple tumour deposits were studied, although large karyotypic differences between the cases were observed. In three cases no karyotypic abnormalities were observed. Smit *et al.* (1990) used DNA FCM to compare DNA indices (DIs) of multiple tumours. In 60% of the cases the similarity in DI provided evidence for metastatic disease. In addition molecular genetic studies provided evidence against a multifocal origin. Mok *et al.* (1992) compared multiple tumours of the same patient for their p53 mutation pattern. In the nine cases investigated the mutations were identical in both tumour sites. Jacobs *et al.* (1992) compared tumours on the basis of LOH at different chromosomes, p53 expression, p53 mutations and X chromosome inactivation pattern. Statistical analysis showed that the probability of an independent occurrence of the tumours was less than 1.5% in 15 of 17 cases.

In the present study we have investigated the clonality of bilateral ovarian carcinomas by DNA FCM and molecular genetic analysis with particular attention to possible clonal divergence after metastasis. To this end we used 36 polymorphic microsatellite markers mapping to eight different chromosomes for LOH analysis. Furthermore, we compared the discriminating power of DNA FCM as a rapid technique with the more laborious approach of LOH analysis. To eliminate the interfering effect of contaminating normal DNA, we applied flow cytometric cell sorting on the basis of DNA content to enrich tumour cell populations for PCR-driven LOH analysis.

Correspondence: ECA Abeln
Received 28 February 1995; revised 31 May 1995; accepted 12 June 1995.

### Materials and methods

### Specimens

Sixteen cases of epithelial ovarian cancer with bilateral tumour locations were used (Table I). Nine were formalin-fixed, paraffin-embedded archival specimens and seven were snap-frozen fresh specimens stored at $-70°C$. Tumour cell percentages were determined by visual examination of haematoxylin- and eosin-stained sections. When specimens contained less than 80% tumour cells, they were enriched by flow cytometric cell sorting (Table I) based on DNA ploidy as described below. Constitutional DNA was extracted from peripheral blood leucocytes. If blood was unavailable, archival paraffin blocks with normal (non-tumour) tissue from the same patient or flow cytometrically sorted, DNA diploid, stromal cells from the aneuploid tumour served as the source for constitutional DNA (Table I).

### Flow cytometry and cell sorting

Suspensions of paraffin-embedded nuclei were obtained according to Hedley *et al.* (1983) with minor modifications (Schueler *et al.*, 1993) and stained with propidium iodide after RNAse treatment. Fresh frozen specimens were processed according to the detergent-trypsin method of Vindelov *et al.* (1983*a*). Trout erythrocytes were used as reference (Vindelov *et al.*, 1983*b*). Samples were analysed on a FACScan flow cytometer (Becton Dickinson, Mountain View, CA, USA). Flow cytometric sorting was performed on the basis of DNA content on a FACStar flow cytometer (Becton Dickinson). DIs were calculated according to accepted criteria (Hiddemann *et al.*, 1984).

**Table I** Histology, specimen type and percentages of tumour cells

| Case | Histology | Specimen type | Tumour a Location | Tumour a Percentage | Tumour b Location | Tumour b Percentage |
|---|---|---|---|---|---|---|
| OV1 | Clear cell | Paraffin | Left ovary | 50[a] | Right ovary | 50[a] |
| OV2 | Endometrioid | Paraffin | Left ovary | 80 | Right ovary | 70 |
| OV3 | Serous | Paraffin | Left ovary | 70 | Right ovary | 80 |
| OV4 | Undifferentiated | Paraffin | Left ovary | 90 | Right ovary | 90 |
| OV5 | Serous | Paraffin | Left ovary | 30[a] | Right ovary | 30[a] |
| OV6 | Endometrioid | Paraffin | Left ovary | 80 | Right ovary | 90 |
| OV7 | Serous | Paraffin | Left ovary | 10[a] | Right ovary | 50[a] |
| OV8 | Endometrioid | Paraffin | Left ovary | 50[a] | Right ovary | 60[a] |
| OV9 | Serous | Paraffin | Left ovary | 60[a] | Right ovary | 40[a] |
| OV10 | Undifferentiated | Frozen | Left ovary | 70 | Right ovary | 50[a] |
| OV11 | Serous | Frozen | Left ovary | 20[a] | Right ovary | 30[a] |
| OV12 | Undifferentiated | Frozen | Left ovary | 80[a] | Right ovary | 60[a] |
| OV13 | Undifferentiated | Frozen | Left ovary | 40[a] | Right ovary | 80 |
| OV14 | Serous | Frozen | Right ovary | 10[a] | Left ovary | 40[a] |
| OV15 | Serous | Frozen | Left ovary | 90[a] | Right ovary | 90[a] |
| OV16 | Serous | Frozen | Left ovary | 70[a] | Right ovary | 90[a] |

[a]Sorted on the basis of DNA ploidy before DNA isolation.

*DNA isolation*

DNA was extracted from peripheral blood lymphocytes according to Miller *et al.* (1988). DNA from fresh tumour specimens was isolated as described by Devilee *et al.* (1989). DNA from paraffin-embedded tissue was isolated by overnight incubation of six 10 μm sections in 100 μl PK buffer [10 mmol $l^{-1}$ Tris-HCl pH 8.3, 0.5% Tween 20, 1 mmol $l^{-1}$ EDTA 1% proteinase K (w/v)] (Limpens *et al.*, 1993), at 56°C followed by a 10 min incubation at 100°C to inactivate proteinase K. To obtain a proper separation of the liquid and solid phases, the tubes were centrifuged directly while samples were hot. The liquid phase was carefully pipetted through the solid paraffin residue laying on top of the sample. DNA was isolated from the sorted nucleus fractions as described before (Abeln *et al.*, 1994). Briefly, nuclear concentrations were adjusted to 50 nuclei per μl with PK buffer and incubated overnight at 56°C followed by a 10 min proteinase K inactivation at 100°C.

*Detection of LOH*

The microsatellite markers that were selected on the basis of heterozygosity and chromosomal location are presented in Table II. At least one marker was used for each chromosome, depending on interpretability and informativeness of the alleles. In non-informative cases, adjacent markers were used. Analysis of LOH was performed by PCR on microsatellite markers as described by Weber and May (1989). Briefly, 2 μl of DNA solution was loaded on a microtitre plate. Reaction mix was added to a total reaction volume of 15 μl containing 3 pmol of the forward and 3 pmol of the reverse primer, 1% bovine serum albumin (BSA), 0.01% gelatin, 0.1% Triton X-100, 10 mmol $l^{-1}$ Tris-HCl pH 9.0, 50 mmol $l^{-1}$ potassium chloride, 1.5 mmol $l^{-1}$ magnesium chloride, 200 μmol $l^{-1}$ dATP, 200 μmol $l^{-1}$ dGTP, 200 μmol $l^{-1}$ dTTP, 0.25 μmol $l^{-1}$ dCTP and 0.1 μl [$\alpha$-$^{32}$P]dCTP (3000 Ci $mmol^{-1}$, 10 μCi $\mu l^{-1}$) (Amersham Nederland, 's-Hertogenbosch, The Netherlands) and 0.06 U of Super *Taq* DNA polymerase (Sphaero Q, HT Biotechnology, Cambridge, UK). Samples were covered with a drop of mineral oil (Sigma, St Louis, MO, USA) and were passed through a temperature cycling programme consisting of 4 min at 94°C, 33 cycles of 1 min at 94°C, 2 min at 55°C, 1 min at 72°C followed by an additional extension step of 6 min at 72°C in a thermocycling machine (MJ Research, Watertown, MA, USA). Samples were denatured by addition of a drop of 0.3% xylene cyanol, 0.3% bromophenol blue, 10 mmol $l^{-1}$ EDTA pH 0.90% (v/v) formamide and subjected to electrophoresis on a 0.4-mm-thick 6.5% polyacrylamide gel containing 7 mol $l^{-1}$ urea. Gels were dried and exposed to X-ray films (Konica, Tokyo, Japan) for periods ranging from 12 to 96 h. Results were interpreted by visual comparison of the allele intensities.

**Table II** Microsatellite markers

| Marker | Chromosomal location | Heterozygosity |
|---|---|---|
| D1S162 | 1p32 | 0.91 |
| D1S175 | 1p21-q12 | 0.80 |
| APOA2 | 1q21-q23 | 0.74 |
| D1S158 | 1q32-q41 | 0.89 |
| D1S103 | 1q32-qter | 0.88 |
| D3S1270 | 3p | 0.75 |
| D3S11 | 3p21-p14 | 0.93 |
| GLUT2 | 3q26-q26.3 | 0.91 |
| D3S1232 | 3q21 | 0.75 |
| IGF2R | 6q25-q27 | 0.42 |
| F13A1 | 6p25-p24 | 0.78 |
| D6S89 | 6p24-p23 | 0.92 |
| D6S251 | 6q13-q21.1 | 0.78 |
| D6S249 | 6q13-q21.1 | 0.46 |
| D11S554 | 11p12-p11.2 | 0.91 |
| D11S875 | 11pter-p11.2 | 0.90 |
| D11S871 | 11pter-p11.2 | 0.77 |
| TP53 | 17p13.1 | 0.68 |
| D17S515 | 17q22-ter | 0.88 |
| D17S520 | 17p12 | 0.77 |
| D17S513 | 17p13 | 0.89 |
| D17S579 | 17q12-q21 | 0.87 |
| D17S250b | 17q11.2-q12 | 0.94 |
| D17S588 | 17q22-23 | 0.68 |
| 46E6 | 17q23-24 | 0.76 |
| HOX2B | 17q21.1-q21.3 | 0.74 |
| D18S40 | 18p11.21-ter | 0.82 |
| D18S35 | 18q11-q12 | 0.72 |
| D18S34 | 18q11 | 0.81 |
| MBP | 18q22-qter | 0.80 |
| D22S156 | 22q11 | 0.78 |
| IL-2RB | 22q11.2-q12 | 0.91 |
| CYP2D | 22q13 | 0.80 |
| KALLMAN | Xp22.3 | 0.61 |
| DXS453 | Xp11.23-q21.1 | 0.72 |
| DXS454 | Xq21.1-q23 | 0.75 |

*Statistics*

The difference (*d*) between the multiple DIs can be caused by a measurement error or by a true biological difference.' Previously Smit *et al.* (1990) derived a likelihood ratio (LR) that determines the probability that *d* is caused by matched pairing *vs* random pairing:

$$LR = \frac{\sigma}{\sigma_e} e^{-1/4\left[\frac{1}{\sigma_e^2} - \frac{1}{\sigma^2}\right]d^2}$$

Where $\sigma_e$ is the measurement error variance of the natural logarithm of the DI (LDI). The calculated value for $\sigma_e$ was 0.041, based on repeated flow cytometric experiments (Kute *et al.*, 1988). $\sigma$ is the variance of LDI of randomly paired DIs of ovarian tumours and was determined to be 0.24 (Smit *et al.*, 1990).

Statistical analysis of the LOH data was performed according to Jacobs *et al.* (1992) with minor modifications. Where *n* deposits of a tumour have loss of heterozygosity for a polymorphism, the probability of each deposit losing the same allele as an independent even is $(1/2)^{n-1}$. This approach does not take the locus-specific chance for allele loss into consideration. In order to do this, chromosome arm-specific LOH frequencies were derived from the pooled data of four ovarian cancer allelotype studies (Sato *et al.*, 1991; Cliby *et al.*, 1993; Yang-Feng *et al.*, 1993; Osborne and Leech, 1994). For a locus $i$ the LOH frequency is $x_i$. If LOH of locus $i$ is observed for tumour a, the probability that LOH occurred at this locus in tumour b independently is $x_i$, and the probability that the same allele is involved is $\frac{1}{2}x_i$. The probability that in a specific case all observed concordant LOH patterns occurred as independent events is equal to the product of the single probabilities and is designated as the probability of independent origin (PIO). In the calculation of the PIOs we used a conservative approach. For example, when multiple markers on the same chromosomes showed LOH (indicating loss of a whole chromosome) only the highest LOH frequency was included in the PIO calculation since these multiple LOH patterns were probably not independent events.

The same was done for cases where identical chromosomal breakpoints were found. It should be considered however, that the probability that chromosomal breakpoints independently occur in the same region in two tumour deposits will probably be lower than the regional LOH frequency.

### Results

### *Flow cytometry*

Nuclear suspensions of all samples were prepared for DNA ploidy analysis. In eight of the 16 cases (OV1, OV2, OV3, OV4, OV6, OV7, OV15 and OV16) the DI was identical in both tumour sites investigated, in five cases (OV8, OV9, OV11, OV12 and OV14) the difference in DI was relatively small (0.1) and in three cases DIs between 0.2 and 0.9 were observed (OV5, OV10 and OV13) (Table III). In Figure 1 the DNA histograms of tumour a and tumour b from case OV14 are shown. Both histograms show an aneuploid population with almost similar DIs (1.7 and 1.6) with varying fractions of diploid cells. The LR of 1.39 calculated for this case is slightly in favour of a unifocal origin. In a total of 14 cases DIs were compatible with a unifocal origin (LR range 1.39–5.85). The extremely low LRs found for cases OV5, OV10 and OV13 made it unlikely that the differences in DI were caused by measurement errors, and thus indicate true biological differences.

### *Detection of LOH*

Samples from 24 out of 32 tumours were enriched for their tumour cell content by flow cytometric cell sorting based on DNA aneuploidy (Table I). The effect of the enrichment is demonstrated in Figure 2. Both sorted diploid fractions of OV11 show two constitutional alleles for microsatellite marker D18S34, while a relative intensity decrease of the upper allele in the unsorted samples was observed, strongly suggesting LOH which is confirmed by PCR amplification of the aneuploid fraction showing a complete loss of the upper allele. The results of comparative LOH analysis of the 16 tumour pairs are summarised in Figure 3. Fourteen cases were informative concerning LOH. The cases were divided into three groups according to concordant or discordant LOH patterns. Group I includes 12 cases showing concordant LOH and ROH patterns for all informative markers. Within this group cases are ranked according to the number of loci showing concordant LOH, ranging from six (case OV13) to zero concordant LOH patterns. Case OV7 was also included in this group although no LOH was detected at any of the investigated loci. For case OV8 no signal was obtained for chromosome 22 in tumour b but concordance was observed for all other markers. The calculated probabilities that the observed LOH patterns could have occurred by coincidental loss of the same allele at all loci ranged from 0.000021 to 0.28 (Table III). Because of the absence of concordant LOH patterns no probabilities could be calculated for cases OV7 and OV8. The LOH data and the calculated probabilities are in most cases highly suggestive for a unifocal origin of the tumours.

Cases in group III show discordance for all observed LOH patterns. Loss of opposite alleles was not observed.

**Table III** Comparison of DNA flow cytometry and LOH-derived probabilities of independent origin

| *Group* | *Case* | $DI_a$ | $DI_b$ | *LR* | *Probability of independent origin* |
|---|---|---|---|---|---|
| I | OV13 | 1.9 | 1.0 | $1.29 \times 10^{-50}$ | 0.000021 |
| | OV2 | 2.0 | 2.0 | 5.85 | 0.00052 |
| | OV16 | 0.9 | 0.9 | 5.85 | 0.00097 |
| | OV12 | 1.8 | 1.7 | 1.39 | 0.0053 |
| | OV15 | 1.3 | 1.3 | 5.85 | 0.0043 |
| | OV1 | 1.6 | 1.6 | 5.85 | 0.050 |
| | OV4 | 1.2 | 1.2 | 5.85 | 0.043 |
| | OV6 | 2.0 | 2.0 | 5.85 | 0.026 |
| | OV14 | 1.7 | 1.6 | 1.39 | 0.060 |
| | OV5 | 1.7 | 1.9 | $18 \times 10^{-3}$ | 0.28 |
| | OV7 | 1.8 | 1.8 | 5.85 | – |
| | OV8 | 1.5 | 1.4 | 1.39 | – |
| II | OV11 | 1.6 | 1.7 | 1.39 | 0.0089 |
| | OV9 | 1.4 | 1.3 | 1.39 | 0.28 |
| III | OV3 | 1.3 | 1.3 | 5.85 | – |
| | OV10 | 1.5 | 1.9 | $5.77 \times 10^{-10}$ | – |

**Figure 1** DNA histograms from two ovarian tumours, located in the left (OV14a) and the right (OV14b) ovary. In both histograms three peaks are visible: trout erythrocytes (tr) used as a ploidy reference, the $G_{1,0}$ from the DNA diploid population (di) and the $G_{1,0}$ from the DNA aneuploid population (an).

**Figure 2** LOH analysis on DNA isolated from tumour fractions enriched by flow cytometry on the basis of DNA ploidy differences. Sorted fractions (di = diploid and an = aneuploid) from both tumours (a and b) were compared with unsorted tumour cell samples (T). Nuclei from case OV11 were isolated. LOH analysis was performed with microsatellite markers: D18S34.

Remarkably, in both cases LOH was found in only one tumour site (OV3b and OV10b).

Group II consists of two cases which showed concordant as well as disconcordant LOH patterns. Figure 4 shows the partial allelotype for four informative microsatellite markers of case OV11. LOH can be observed in the aneuploid fraction of tumour a as well as in the aneuploid fraction of tumour b for D17S588 since in both lanes the upper allele is completely lost, while ROH was observed for marker D3S1270. However, only the aneuploid fraction of tumour a shows LOH for the chromosome 6 markers F13A1 and D6S251 while ROH for tumour b was observed. The markers for the other chromosomes all showed concordant patterns (LOH for chromosomes 17, 18p and 22 and ROH for chromosomes 3, 11, 18q and X). Together with identical chromosome 18 breakpoints in both tumours (between D18S34 and D18S40) this strongly suggests a unifocal origin. This is supported by the calculated PIO of 0.0089. Although in case OV9 an independent origin cannot be excluded on the basis of a PIO of 0.28, the presence of identical chromosome

**Figure 3** Graphic representation of the allelotypes of the ovarian tumours for chromosomes 1, 3, 6, 11, 17, 18, 22 and X. The left chromosome of each chromosome pair corresponds to tumour a and the right chromosome to tumour b of each case. The small numbers indicate the number of informative markers for each chromosome. Black symbols indicate LOH, white symbols ROH. When microsatellite markers could not properly be amplified or where all tested markers were homozygous for a specific chromosome no chromosome symbol was drawn. Case numbers are indicated at the left side. The cases are grouped according to the occurrence of concordant LOH patterns. Within the groups the cases were ranked according to descending number of observed LOH events.

**Figure 4** Partial allelotype of case OV11. Four different DNA samples were amplified with microsatellite markers D3S1270, D6S251, F13A1 and D17S588. LOH can be observed in the aneuploid fractions of tumour a and tumour b with marker D17S588 and retention with marker D3S1270. For the microsatellite markers located on chromosome 6 (D6S251 and F13A1) only tumour a has lost an allele while tumour b shows retention of heterozygosity.

17 breakpoints, identified by four informative markers is still strongly suggestive of a unifocal origin of both tumours.

*LOH* vs *DNA index*

In group I identical allelotypes correlate to equal or nearly equal DIs ($d = <0.1$) in all cases except OV5 and OV13. However, the PIO values show a much higher variation than the LRs for these cases. Although, for OV13 the LOH patterns are totally identical for all informative markers on seven different chromosomes (PIO = 0.000021), a large difference in DI was observed (LR = $1.29 \times 10^{-50}$). This discrepancy can be explained by assuming that the aneuploid stemline in tumour a represents a tetraploidised subclone of the diploid stemline of tumour b. This subclone must have originated after the establishment of the observed allelotype.

In case OV5 neither DNA ploidy analysis nor the allelotype can exclude an independent origin. Although both tumours lost the complete chromosome 17 this still could be an independent event since loss of chromosome 17 is very common in ovarian tumours. Despite a non-complete identity of the allelotypes of the two cases in group II the FCM results are also in favour of a unifocal origin of the tumours. In group III the discordant LOH patterns correlate with differences in DNA index for case OV10 while the two methods gave contradictory results for OV3.

### Discussion

In the present study two different genetic approaches were used to study the origin of bilateral ovarian tumours. We found evidence for a unifocal origin in 14 of 16 cases on the basis of LOH analysis, whereas DNA ploidy analysis did not always provide conclusive evidence about the origin of tumours. The use of DNA ploidy analysis is based on the assumption that identical, aneuploid DIs are not likely to be the product of an independent ploidy evolution but rather reflect a unifocal origin. DNA FCM is a rapid technique which is used in many clinical pathological laboratories throughout the world. A limitation to this type of analysis is that ploidy evolution may continue after the formation of metastases, e.g. by tetraploidisation. This would result in a DI difference that might be interpreted as evidence against a unifocal origin. An illustration of this phenomenon is case OV13 in which DIs of 1.9 and 1.0 were observed while the allelotype was completely identical including LOH at six different chromosomes (Figure 3). Tetraploidisation apparently occurred after establishment of the allelotype. Also in the case of DI heterogeneity in the primary tumour metastasis of a minor subpopulation not detected in the primary tumour might yield a similar DI difference. Apart from this the limited resolution of DNA FCM which on average cannot detect DNA content differences less than 5% may lead to spurious identity of DIs.

Microsatellite-based LOH analysis can detect genetic differences at the subchromosomal level and is intrinsically more sensitive and specific. In contrast to DNA ploidy analysis, ongoing clonal evolution at different tumour locations might not preclude the identification of a unifocal origin owing to the clonal retention of acquired genetic aberrations, e.g. the two cases OV9 and OV11 in group II. The specificity of this approach depends on the number of independent LOH events as well on the a priori probability that a certain chromosome region will show LOH, e.g. regions harbouring tumour-suppressor genes. For instance, a coincidental, identical LOH pattern for chromosome 17 will be more likely than for chromosome 1. We have corrected for this in our calculations. With the present availability of highly polymorphic micro-satellite markers, the accurate mapping of chromosomal breakpoints can substantially increase the power of this type of analysis and provide definite answers about the clonality of multiple tumours. Identical chromosomal breakpoints are a strong indication of a unifocal origin. The accuracy by which the exact position of breakpoints and thus their identity can be determined, depends on the proximity of the flanking microsatellite markers. Other PCR-based methods that can provide information about the origin of multiple tumours are determination of X-chromosome inactivation (Allen *et al*., 1992), oncogene amplification (Li *et al*., 1994) and determination of oncogene or tumour-suppressor gene mutations. The use of X chromosome inactivation is hampered by the fact that half of the multifocal tumours will have identical inactivation just by chance. Oncogene amplification is less informative because (1) a limited number of chromosomal regions is known to be involved and (2) certain amplicons are strongly correlated with certain tumour types which makes an independent amplification in both tumours rather likely and therefore less appropriate. Mutation analysis of frequently involved oncogenes or tumour-suppressor genes like p53 is highly informative and applicable in many kinds of tumours including ovarian tumours. Disadvantages are the lack of mutations in a high percentage of tumours (for ovarian tumours about 50%), presence of mutational hot spots (*ras*, p53) and the possibility that a mutation occurs after metastatic spread.

A limitation of LOH analysis on DNA extracted from total tumour tissue is the fraction contaminating non-neoplastic cells. Under optimal conditions the minimum amount of tumour cells should exceed 40% (Gruis *et al.*, 1993). We recently showed that tumour specimens with lower amounts of neoplastic cells can be made accessible for PCR-based LOH analysis by flow sorting of tumour cells on the basis of DNA ploidy and marker expression (Abeln *et al.*, 1994). In the present study this enabled us to successfully enrich 24 of the 32 tumours where otherwise 18 tumours would have been excluded owing to low tumour cellularity.

In 10 of the 16 investigated cases statistical evidence of unifocal origin was found on the basis of LOH and in two cases (OV5 and OV9) evidence was obtained which suggested unifocal origin but failed to reach the level of statistical significance. For case OV9 the identical chromosome 17 breakpoint in both tumours provides strong evidence for a common origin of the different tumour sites. These findings are in agreement with previous reports based on DNA flow cytometry, karyotyping, p53 mutation analysis or allelotyping (Smit *et al.*, 1990; Pejovic *et al.*, 1991; Jacobs *et al.*, 1992; Mok *et al.*, 1992) in which 31/43, 11/11, 9/9 and 15/17 respectively, showed evidence for unifocal origin.

From a theoretical point of view formal proof that tumours are multifocal is quite difficult. Only if the initial molecular event is known (as in e.g. APC mutation in colorectal adenoma development; Powell *et al.*, 1992) discordance for this marker will be direct proof of multifocality. The tumours in group III showed discordant LOH which suggests but does not prove multifocality since post-metastatic LOH does occur as evidenced by the tumours in group II.

The PIO calculation is correct under the condition that LOH is non-parental origin-specific. If a certain chromosomal region harbouring a tumour-suppressor gene is imprinted, loss of the other allele may totally switch off its suppressor function and will provide selective advantage with regard to carcinogenesis. The chance that two tumours in a patient independently lose the non-imprinted allele therefore may be higher than in a non-imprinted situation. In this case the PIO would be a less accurate measure for independent origin. The same holds for cases where LOH is the second hit uncovering a germline mutation according to Knudson's two-hit tumour-suppressor gene model, but this will be restricted to familial tumours. Parental specific LOH has been reported for some cases (reviewed by Feinberg, 1993) concerning Wilms' tumour, rhabdomyosarcoma, bilateral retinoblastoma, unilateral retinoblastoma, acute myelogenous leukaemia and neuroblastoma concerning regions on chromosomes 1, 2, 7, 11 and 13. However this phenomenon has not been reported for ovarian cancer, although LOH at chromosome 11 is a frequent event. However both for the imprinted as well as in familial tumours, partial LOH generating a chromosomal breakpoint, would still enable discrimination of multifocal from unifocal origin.

In our series chromosomes 6, 11, 17 and 18 were most frequently involved in LOH. LOH at 6q was previously reported to be a common event (51%) in ovarian tumours (Saito *et al.*, 1992), although a candidate tumour-suppressor gene has not yet been reported in this region. WT1 may be a target gene for the loss of chromosome 11 (Eccles *et al.*, 1992; Foulkes *et al.*, 1993). TP53 could be the target gene for the chromosome 17p loss, since the gene is frequently involved in ovarian cancer (Milner *et al.*, 1993). For *BRCA1* which also maps to chromosome 17, recently both germline (Futreal *et al.*, 1994) as well as somatic mutations have been found in ovarian cancer (Hosking *et al.*, 1995; Merajver *et al.*, 1995) although a second gene on 17q may be a more likely target (Saito *et al.*, 1993). Frequent LOH at 18q (60%) has also previously been reported, however the smallest region of overlap seems to exclude the *DCC* tumour-suppressor gene (Chenevix-Trench *et al.*, 1992).

Interestingly two cases, OV9 and OV11, showed discordant LOH patterns for chromosomes 6 and 18 and concordance was observed at other loci. In contrast, relatively high LOH frequencies for chromosomes 6 (62%) and 18 (43%) were reported by Cliby *et al.* (1993). Our results suggest that LOH at chromosomes 6 and 18 may represent late events in tumour progression which in some tumours may not be essential for establishing the malignant phenotype. Whether the absence of LOH at chromosomes 6 and 18 respectively, in one of the sites definitely identifies these as the primary tumours is still unclear. Evidence for clonal divergence was previously reported by Jacobs *et al.* (1992) although in this study fewer loci were investigated.

Our results support the evidence for a monoclonal origin of the majority of bilateral ovarian carcinomas. PCR-based LOH analysis proved to be a more reliable technique for clonality determination than DNA FCM. The results obtained with both techniques indicate that clonal divergence after metastasis is an infrequent phenomenon in ovarian cancer.

**Acknowledgements**
We are grateful to Jan Beentjes from the Department of Radiology from the academic hospital in Leiden for his photographic assistance and Elly Fieret from the Dr Daniel den Hoed Cancer Center in Rotterdam for technical assistance. We also want to thank Dr IJ Jacobs from the University of Cambridge for statistical advice. Microsatellite markers were obtained from the Dutch Microsatellite Marker Bank, a project supported by NWO (The Dutch Foundation for Scientific Research).

**References**

ABELN ECA, CORVER WE, KUIPERS-DIJKSHOORN NJ, FLEUREN G-J AND CORNELISSE CJ. (1994). Molecular genetic analysis of flow-sorted ovarian tumour cells: improved detection of loss of heterozygosity. *Br. J. Cancer*, **70**, 255–262.

ALLEN CR, ZOGHBI HY, MOSELY AB, ROSENBLATT HM AND BELMONT JW. (1992). Methylation of HpaII and HhaI sites near the polymorphic CAG repeat in the human androgen-receptor gene correlates with X chromosome inactivation. *Am. J. Hum. Genet.*, **51**, 1229–1239.

BONSING BA, DEVILEE P, CLETON-JANSEN A-M, KUIPERS-DIJKSHOORN NJ, FLEUREN GJ AND CORNELISSE CJ. (1993). Evidence for limited molecular genetic heterogeneity as defined by allelotyping and clonal analysis in nine metastatic breast carcinomas. *Cancer Res.*, **53**, 3804–3811.

BORING CC, SQUIRES TS AND TONG T. (1991). Cancer statistics, 1991. *CA*, **41**, 19–36.

CHENEVIX-TRENCH G, LEARY J, KERR J, MICHEL J, KEFFORD R, HURST T, PARSONS PG, FRIEDLANDER M AND KHOO SK. (1992). Frequent loss of heterozygosity on chromosome 18 in ovarian adenocarcinoma which does not always include the DCC locus. *Oncogene*, **7**, 1059–1065.

CLIBY W, RITLAND S, HARTMANN L, DODSON M, HALLING KC, KEENEY G, PODRATZ KC AND JENKINS RB. (1993). Human epithelial ovarian cancer allelotype. *Cancer Res.*, **53**, 2393–2398.

DEVILEE P, VAN DEN BROEK M, KUIPERS-DIJKSHOORN N, KOLLURI R, KHAN PM, PEARSON PL AND CORNELISSE CJ. (1989). At least four different chromosomal regions are involved in loss of heterozygosity in human breast carcinoma. *Genomics*, **5**, 554–560.

ECCLES DM, GRUBER L, STEWART M, STEEL CM AND LEONARD RCF. (1992). Allele loss on chromosome 11p is associated with poor survival in ovarian cancer. *Disease Markers*, **10**, 95–99.

FEINBERG AP. (1993). Genomic imprinting and gene activation in cancer. *Nature Genet.*, **4**, 110-113.

FOULKES WD, CAMPBELL IG, STAMP GW AND TROWSDALE J. (1993). Loss of heterozygosity and amplification on chromosome 11q in human ovarian cancer. *Br. J. Cancer*, **67**, 268-273.

FUTREAL PA, LIU Q, SHATTUCK-EIDENS D, COCHRAN C, HARSHMAN K, TAVTIGIAN S, BENNETT LM, HAUGEN-STRANO A, SWENSEN J, MIKI Y, EDDINGTON K, McCLURE M, FRYE C, WEAVER-FELDHAUS J, DING W, GHOLAMI Z, SÖDERKVIST P, TERRY L, JHANWAR S, BERCHUCK A, IGLEHART JD, MARKS J, BALLINGER DG AND BARRETT JC. (1994). *BRCA1* mutations in primary breast and ovarian carcinomas. *Science*, **266**, 120-122.

GRUIS NA, ABELN ECA, BARDOEL AFJ, DEVILEE P, FRANTS RR AND CORNELISSE CJ. (1993). PCR-based microsatellite polymorphisms in the detection of loss of heterozygosity in fresh and archival tumour tissue. *Br. J. Cancer*, **68**, 308-313.

HEDLEY DW, FRIEDLANDER ML, TAYLOR IW, RUGG CA AND MUSGROVE EA. (1983). Method for analysis of cellular DNA content of paraffin-embedded pathological material using flow cytometry. *J. Histochem. Cytochem.*, **31**, 1333-1335.

HIDDEMANN W, SCHUMANN J, ANDREEF M, BARLOGIE B, HERMAN CJ, LEIF RC, MAYALL BH, MURPHY RF AND SANDBERG AA. (1984). Convention on nomenclature for DNA cytometry. Committee on Nomenclature, Society for Analytical Cytology. *Cancer Genet. Cytogenet.*, **13**, 181-183.

HOSKING L, TROWSDALE J, NICOLAI H, SOLOMON E, FOULKES W, STAMP G, SIGNER E AND JEFFREYS A. (1995). A somatic *BRCA1* mutaion in an ovarian tumour. *Nature Genet.*, **9**, 343-344.

JACOBS IJ, KOHLER MF, WISEMAN RW, MARKS JR, WHITAKER R, KERNS BAJ, HUMPHREY P, BERCHUCK A, PONDER BAJ AND BAST RC Jr. (1992). Clonal origin of epithelial ovarian carcinoma: analysis by loss of heterozysity, p53 mutation, and X-chromosome inactivation. *J. Natl Cancer Inst.*, **84**, 1793-1798.

KUTE TE, GREGORY B, GALLESHAW J, HOPKINS M, BUSS D AND CASE D. (1988). How reproducible are flow cytometry data from paraffin-embedded blocks? *Cytometry*, **9**, 494-498.

LI BDL, HARLOW SP, BUDNICK RM, SHEEDY DL AND CARLETON CC. (1994). Detection of HER-2/neu oncogene amplification in flow cytometry sorted breast ductal cells by competitive polymerase chain reaction. *Cancer*, **73**, 2771-2778.

LIMPENS J, BEELEN M, STAD R, HAVERKORT M, VAN KRIEKEN JH, VAN OMMEN GJ AND KLUIN PM. (1993). Detection of the t(14;18) translocation in frozen and formalin-fixed tissue. *Diagn. Mol. Pathol.*, **2**, 99-107.

MERAJVER SD, PHAM TM, CADUFF R, POY EL, COONEY KA, WEBER BL, COLLINS FS, JOHNSTON C AND FRANK TS. (1995). Somatic mutations in the BRCA1 gene in sporadic ovarian tumours. *Nature Genet.*, **9**, 439-450.

MILLER SA, DYKES DD AND POLESKY HF. (1988). A simple salting out procedure for extracting DNA from human nucleated cells. *Nucleic Acids Res.*, **16**, 1215.

MILNER BJ, ALLAN LA, ECCLES DM, KITCHENER HC, LEONARD RCF, KELLY KF, PARKIN DE AND HAITES NE. (1993). *p53* mutation is a common genetic event in ovarian carcinoma. *Cancer Res.*, **53**, 2128-2132.

MOK CH, TSAO SW, KNAPP RC, FISHBAUGH PM AND LAU CC. (1992). Unifocal origin of advanced human epithelial ovarian cancers. *Cancer Res.*, **52**, 5119-5122.

NOVAK ER AND WOODRUFF JD. (1974). *Novak's Gynecologic and Obstetric Pathology*, pp. 367-393. WB Saunders: Philadelphia.

OSBORNE RJ AND LEECH V. (1994). Polymerase chain reaction allelotyping of human ovarian cancer. *Br. J. Cancer*, **69**, 429-438.

PEJOVIC T, HEIM S, MANDAHL N, ELMFORS B, FURGYIK S, FLODERUS UM, HELM G, WILLEN H AND MITELMAN F. (1991). Bilateral ovarian carcinoma: cytogenetic evidence of unicentric origin. *Int. J. Cancer*, **47**, 358-361.

POWELL SM, ZILZ N, BEAZER-BARCLAY Y, BRYAN TM, HAMILTON SR, THIBODEAU SN, VOGELSTEIN B AND KINZLER KW. (1992). *APC* mutations occur early during colorectal tumorigenesis. *Nature*, **359**, 235-237.

SAITO S, SAITO H, KOI S, SAGAE S, KUDO R, SAITO J, NODA K AND NAKAMURA Y. (1992). Fine-scale deletion mapping of the distal long arm of chromosome 6 in 70 human ovarian cancers. *Cancer Res.*, **52**, 5815-5817.

SAITO H, INAZAWA J, SAITO S, KASUMI F, KOI S, SAGAE S, KUDO R, SAITO J, NODA K AND NAKAMURA Y. (1993). Detailed deletion mapping of chromosome 17q in ovarian and breast cancers: 2-cM region on 17q21.3 often and commonly deleted in tumours. *Cancer Res.*, **53**, 3382-3385.

SATO T, SAITO H, MORITA R, KOI S, LEE JH AND NAKAMURA Y. (1991). Allelotype of human ovarian cancer. *Cancer Res.*, **51**, 5118-5122.

SCHUELER JA, CORNELISSE CJ, HERMANS J, TRIMBOS JB, VAN DER BURG MEL AND FLEUREN GJ. (1993). Prognostic factors in well-differentiated early-stage epithelial ovarian cancer. *Cancer*, **71**, 787-795.

SMIT VTHBM, FLEUREN GJ, VAN HOUWELINGEN JC, ZEGVELD ST, KUIPERS-DIJKSHOORN NJ AND CORNELISSE CJ. (1990). Flow cytometric DNA-ploidy analysis of synchronously occurring multiple malignant tumors of the female genital tract. *Cancer*, **66**, 1843-1849.

VINDELOV LL, CHRISTENSEN IJ AND NISSEN NI. (1983*a*). A detergent-trypsin method for the preparation of nuclei for flow cytometric DNA analysis. *Cytometry*, **3**, 323-327.

VINDELOV LL, CHRISTENSEN IJ AND NISSEN NI. (1983*b*). Standardization of high-resolution flow cytometric DNA analysis by the simultaneous use of chicken and trout red blood cells as internal reference standards. *Cytometry*, **3**, 328-331.

WEBER JL AND MAY PE. (1989) Abundant class of human DNA polymorphisms which can be typed using the polymerase chain reaction. *Am. J. Hum. Genet.*, **44**, 388-396.

YANG-FENG TL, HAN H, CHEN K-C, LI S, CLAUS EB, CARCANGIU ML, CHAMBERS SK, CHAMBERS JT AND SCHWARTZ PE. (1993). Allelic loss in ovarian cancer. *Int. J. Cancer*, **54**, 546-551